# Rotational thrombelastometry: a step forward to safer patient care?

**DOI:** 10.1186/s13054-014-0706-7

**Published:** 2014-12-26

**Authors:** Fuat H Saner

**Affiliations:** Department of General, Visceral and Transplant Surgery, Medical Center University of Essen/Germany, Hufelandstr. 55, 45147 Essen, Germany

## Abstract

The study by Hincker and colleagues indicated that the perioperative use of rotational thrombelastometry (ROTEM™) could predict thromboembolic events in 90% of the cases in non-cardiac surgery. Viscoelastic tests (VETs) - ROTEM™ and thrombelastography (TEG™) - are used mainly to predict bleeding complications. Most conventional coagulation tests, like prothrombin time and activated partial thromboplastin time, can identify a disturbance in plasmatic hemostasis. However, the relevance of these assays is limited to the initiation phase of coagulation, whereas VETs are designed to assess the whole clotting kinetics and strength of the whole blood clot and reflect more the interaction between procoagulants, anticoagulants, and platelets. The first reports about VET and hypercoagulable state were published more than 25 years ago. Since then, several studies with different quality and sample size have been published, sometimes with conflicting results. A systematic review about hypercoagulable state and TEG™ indicated that further studies are needed to recommend VETs as a screening tool to predict postoperative thrombosis.

In a previous issue of *Critical Care*, Hincker and colleagues [1] identified with preoperative rotational thrombelastometry (ROTEM™, TEM International, München, Germany) analysis patients at high risk for postoperative thromboembolic events. Viscoelastic tests (VETs) were developed primarily to detect coagulopathy rather than thrombosis. Hartert [2] established thrombelastography (TEG™, Haemonetics, Braintree, MA, US) in 1948. Since that time, TEG™ has had different periods of popularity but has never been routinely used for perioperative coagulation management, except for liver transplantation in Pittsburgh in the 1980s [3].

ROTEM™ is a computerized point-of-care system, similar to TEG™ technologies, but its measurements are more robust than those of TEG™, which enables ROTEM™ for a mobile bedside testing (for example, in the operation theatre or intensive care unit).

Bleeding and blood transfusion are associated with increased mortality and morbidity [4]. VETs are able to predict bleeding complications and to provide a goal-directed coagulation treatment with fibrinogen concentrate, crypoprecipitate, prothrombin complex, platelets, and antifibrinolytic therapy instead of blind fresh frozen plasma (FFP) transfusions, and this treatment avoids negative side effects of FFPs, like transfusion-associated lung injury, transfusion-associated circulation overload, or infections [5]. Other benefits of ROTEM™ are the shorter turnaround time (10 to 15 minutes [5]) compared with conventional coagulation tests (45 to 90 minutes [6,7]).

Akay and colleagues [8] evaluated the efficacy of ROTEM™ to detect hypercoagulopathy in cancer patients compared with healthy controls. The authors indicated that in all four tests - extrinsic thrombelastometry, intrinsic thrombelastometry, fibrinogen thrombelastometry, and aprotinin thrombelastometry - the clot formation time was significantly shorter and maximum clot formation was significant higher compared with healthy controls, indicating a risk for thrombosis. However, there were some problems putting these findings into a clinical context; for example, no data about the incidence of thromboembolic events were provided.

In a cohort study, McCrath and colleagues [9] investigated 240 consecutive patients scheduled for non-cardiac surgery, to identify patients with increased risk for thrombosis with TEG™. The patients were stratified in two groups; those with a maximum amplitude (MA) of greater than 68 mm were assigned as hypercoagulable, and those with an MA of not more than 68 mm were assigned as normal. Thromboembolic complications in patients with an MA of greater than 68 mm were significantly higher compared with those with an MA of not more than 68 mm (8.4% versus 1.4%, *P* = 0.0157). Myocardial infarction occurred only in patients with an increased MA of greater than 68 mm.

Cerutti and colleagues [10] described a TEG™ detected hypercoagulable state in adult living donors, despite decreased platelet count, increased international normalized ratio, and normal activated partial thromboplastin time.

However, some other reports did not find a correlation between hypercoagulability identified by TEG™ and postoperative thrombotic complication [11,12]. Dai and colleagues [13] conducted a meta-analysis comparing several studies performed with TEG™, supposing that TEG™ may be useful to predict thromboembolic events postoperatively. However, because TEG™ technologies have changed over the last 30 years, there is wide variability in TEG™ results in the different studies. As opposed to TEG™ measurements, ROTEM™ measurements are more robust and have an automated pipette, resulting in more reproducible and precise results [14].

## Outlook for the future

Although standard laboratory tests are poor predictors for both bleeding and thrombosis, clinicians or laboratory physicians are very reluctant to use VETs for global assessment for bleeding or thrombosis. Although a Cochrane meta-analysis [15] failed to show that the use of TEG™ or ROTEM™ reduces mortality, at least one prospective randomized trial shows that coagulation management-guided ROTEM™ reduces blood loss and thromboembolic events [16]. The study by Hincker and colleagues [1] shows very encouraging results that ROTEM™ can predict thrombotic complications in non-cardiac surgery. These data should urge us to use more VETs for both bleeding and risk for thrombosis, although these data should be confirmed in a prospective randomized trial.

## Abbreviations

FFP: Fresh frozen plasma
MA: Maximum amplitude
ROTEM™: Rotational thrombelastometry
TEG™: Thrombelastography
VET: Viscoelastic test
